# Increased reproductive tract infections among secondary school girls during the COVID-19 pandemic: associations with pandemic-related stress, mental health, and domestic safety

**DOI:** 10.1093/sexmed/qfae045

**Published:** 2024-07-23

**Authors:** Supriya D Mehta, Debarghya Nandi, Fredrick Otieno, Garazi Zulaika, Elizabeth Nyothach, Walter Agingu, Runa Bhaumik, Linda Mason, Anna Maria van Eijk, Penelope A Phillips-Howard

**Affiliations:** Division of Infectious Diseases, Department of Medicine, Rush University College of Medicine, Chicago, IL 60612, United States; Division of Epidemiology and Biostatistics, School of Public Health, University of Illinois Chicago, Chicago, IL 60612, United States; Division of Epidemiology and Biostatistics, School of Public Health, University of Illinois Chicago, Chicago, IL 60612, United States; Nyanza Reproductive Health Sciences, Kisumu 40100, Kenya; Department of Clinical Sciences, Liverpool School of Tropical Medicine, Liverpool L35QA, United Kingdom; Kenya Medical Research Institute, Kisumu 40100, Kenya; Nyanza Reproductive Health Sciences, Kisumu 40100, Kenya; Division of Epidemiology and Biostatistics, School of Public Health, University of Illinois Chicago, Chicago, IL 60612, United States; Department of Clinical Sciences, Liverpool School of Tropical Medicine, Liverpool L35QA, United Kingdom; Department of Clinical Sciences, Liverpool School of Tropical Medicine, Liverpool L35QA, United Kingdom; Department of Clinical Sciences, Liverpool School of Tropical Medicine, Liverpool L35QA, United Kingdom

**Keywords:** COVID-19, reproductive tract infections, bacterial vaginosis, sexually transmitted infections, adolescent girls and young women, Kenya, cohort, mediation

## Abstract

**Background:**

Kenya, like many countries, shuttered schools during COVID-19, with subsequent increases in poor mental health, sexual activity, and pregnancy.

**Aim:**

We sought to understand how the COVID-19 pandemic may mediate the risk of reproductive tract infections.

**Methods:**

We analyzed data from a cohort of 436 secondary schoolgirls in western Kenya. Baseline and 6-, 12-, and 18-month study visits occurred from April 2018 to December 2019 (pre–COVID-19), and 30-, 36-, and 48-month study visits occurred from September 2020 to July 2022 (COVID-19 period). Participants self-completed a survey for sociodemographics and sexual activity and provided self-collected vaginal swabs for bacterial vaginosis (BV) testing, with sexually transmitted infection (STI) testing at annual visits. We hypothesized that greater COVID-19–related stress would mediate risk via mental health, feeling safe inside the home, and sexual exposure, given the pandemic mitigation–related impacts of school closures on these factors. COVID-19–related stress was measured with a standardized scale and dichotomized at the highest quartile. Mixed effects modeling quantified how BV and STI changed over time. Longitudinal mediation analysis quantified how the relationship between COVID-19 stress and increased BV was mediated.

**Outcomes:**

Analysis outcomes were BV and STI.

**Results:**

BV and STI prevalence increased from 12.1% and 10.7% pre–COVID-19 to 24.5% and 18.1% during COVID-19, respectively. This equated to 26% (95% CI, 1.00–1.59) and 36% (95% CI, 0.98–1.88) higher relative prevalence of BV and STIs in the COVID-19 vs pre–COVID-19 periods, adjusted for numerous sociodemographic and behavioral factors. Higher COVID-19–related stress was associated with elevated depressive symptoms and feeling less safe inside the home, which were each associated with a greater likelihood of having a boyfriend. In mediation analyses, the direct effect of COVID-19–related stress on BV was small and nonsignificant, indicating that the increased BV was due to the constellation of factors that were affected during the COVID-19 pandemic.

**Clinical Translation:**

These results highlight factors to help maintain reproductive health for adolescent girls in future crises, such as anticipating and mitigating mental health impacts, domestic safety concerns, and maintaining sexual health services.

**Strengths and Limitations:**

Impacts of the COVID-19 pandemic on drivers of reproductive tract health among those who did not attend school or who live in different settings may differ.

**Conclusions:**

In this cohort of adolescent girls, BV and STIs increased following COVID-19–related school closures, and risk was mediated by depressive symptoms and feeling less safe in the home, which led to a higher likelihood of sexual exposures.

## Introduction

The World Health Organization declared the end of the COVID-19 pandemic as a public health emergency on May 5, 2023. To help stem the initial impact of COVID-19, Kenya, like many countries around the world, shuttered schools on March 16, 2020, and put in place restrictions and curfews related to travel and social distancing.^1^ Schools were partially reopened on October 19, 2020, and fully reopened on January 4, 2021. The closure of schools, curfews, and restrictions on travel and public gatherings were costly to the economy and disruptive to the social fabric of Kenyans. The World Bank reported that an estimated 2 million Kenyans were newly forced into poverty due to the pandemic.^2^ The tangible impacts of the economic decline led to increases in job loss and food insecurity, especially during the first 6 months of the pandemic, and this fallout extended to many low- and middle-income countries.^3^

During the school closures, relative to pre–COVID-19, there were increases in adolescent pregnancy and a 3-fold higher risk of school dropout observed in our cohort of secondary schoolgirls in western Kenya.^4^ This was a global phenomenon: school closures due to COVID-19 led to permanent school dropout stemming from exacerbated and expanded poverty; added responsibilities of employment, domestic work, and childcare; and pregnancy and marriage.^5^ In meta-analyses of worldwide data, there were increases in depressive symptoms and anxiety among children and adolescents, with greater prevalence and severity seen among girls and older children.^6^^,^^7^ Evidence also emerged showing increases in sexual offenses against children coincident with lockdowns, curfews, and school closures.^8^

Information is emerging on the impact of the COVID-19 pandemic on rates of sexually transmitted infections (STIs), which disproportionately affect adolescent girls and young women (AGYW). While there were some decreases in STIs during the initial lockdown phases, likely due to underreporting and limited access to services,^9^^,^^10^ STIs rebounded at increasing rates over the pre–COVID-19 period in the United States,^11^^,^^12^ China,^13^ and Spain.^14^ Maintaining reproductive tract health is a public health and clinical priority. Bacterial vaginosis (BV) increases the risk of HIV acquisition 1.6-fold,^15^ and nonulcerative STIs raise the risk of HIV acquisition 3- to 5-fold.^16^ This is especially relevant in western Kenya, where the prevalence of HIV in the general adult population ranges from 16% (Kisumu County) to 21% (Siaya County).^17^ BV and STIs are also associated with greater risk of adverse pregnancy outcomes,^18^ which already occur at higher rates among AGYW.^19^

In this analysis, we describe the prevalence of reproductive tract infections in a cohort of Kenyan secondary schoolgirls before, during, and after the COVID-19 pandemic. We also sought to understand whether the COVID-19 pandemic mediated the subsequent risk of reproductive tract infections. We hypothesized that greater COVID-19–related stress would mediate risk via mental health, safety in the home, and sexual exposure, given the pandemic mitigation–related impacts of school closures on these factors. Quantifying the extent to which these factors contribute to reproductive tract infection risk can help with decision making around intervention development and prioritization.

## Methods

This study was approved by the institutional review boards of the Kenya Medical Research Institutes Scientific Ethics Review Unit (3215), Maseno University Ethics Review Committee (MSU/DRPI/MUERC/01021/21), Liverpool School of Tropical Medicine (15-005), and University of Illinois at Chicago (2017-1301). Written informed consent was obtained for all participants, with written assent and guardian consent obtained for nonemancipated minors.

### Study design and participants

Data for this analysis came from the Cups and Community Health (CaCHe) study,^20^^,^^21^ a subset of participants within the Cups or Cash for Girls (CCG) trial (ClinicalTrials.gov: NCT03051789). The CCG trial has been described in detail.^22^ Briefly, CCG was a cluster-randomized controlled trial in which secondary schools were randomized into 4 arms (1:1:1:1): provision of menstrual cups with training on safe cup use and care, conditional cash transfer based on *>*80% school attendance in the previous term, menstrual cup and conditional cash transfer, and usual practice. For the CaCHe study, we enrolled approximately 20% of girls in the cup-only and control arms of the CCG trial. The CaCHe study was powered to detect a 25% reduced relative prevalence of BV for the cup arm as compared with the control arm over 6 study visits. After enrollment, CaCHe participants were followed every 6 months^20^ (Figure S1). The 24-month study visit scheduled to occur May 2020 was missed due to the COVID-19 pandemic. In the intention-to-treat analysis, over 30 months of observation, participants randomized to the menstrual cup arm had a 24% decreased odds of BV as compared with the usual practice arm.^20^ Accordingly, menstrual cups were provided to all usual practice participants after the 30-month visit. The 42-month visit was missed due to a gap in funding.

### Data collected

At each study visit, participants undertook a self-completed survey that collected information on sociodemographics, sexual practices, mental health, and menstrual practices. Participants were asked if they were sexually active in the past 6 months and if they had been coerced or tricked to have sex in the past 6 months. Transactional sex was assessed through a series of questions that assessed sex in exchange for things (eg, pads, money, school fees) or treatment (eg, favors, employment). Few participants reported being married until the 48-month visit (Table 1); thus, the variable used for analysis is referred to as having a “boyfriend” for simplicity. At the 12-, 30-, 36-, and 48-month visits, depressive symptoms were assessed with the 9-item Personal Health Questionnaire (PHQ-9); scores were dichotomized at ≥5 for analyses, reflecting mildly elevated depressive symptoms.^23^ The 12-month visit PHQ-9 score was applied at the baseline and 6- and 18-month visits for longitudinal modeling due to a lack of measure at these time points. Domestic safety was assessed at each time point during the COVID-19 period with a single question: “Since the curfews and school closures due to COVID-19, do you feel more safe or less safe inside your home?” with responses of *less safe*, *the same*, and *more safe*. For analysis, responses were dichotomized as *less safe* vs *the same or more safe*.

**Table 1:** Distribution of participant characteristics by study time point.^a^

	**May 2–Jul 6, 2018**	**Oct 1–Nov 12, 2018**	**May 8–Jul 19, 2019**	**Oct 3-25, 2019**	**Sep 30–Dec 18, 2019 (94%)**	**Apr 25–Jun 11, 2020**	**Apr 5–Jul 2, 2022**
**Variable** ^**b**^	**Baseline (n = 436)**	**6 mo** ^**b**^ **(n = 424)**	**12 mo (n = 395)**	**18 mo (n = 398)**	**30 mo (n = 395)**	**36 mo (n = 329)** ^**c**^	**48 mo (n = 364)**
Age, y	16.9 (16.1-17.9)	17.4 (16.5-18.3)	17.9 (17.1-18.6)	18.2 (17.4-19.2)	19.4 (18.5-20.4)	20.0 (19.0-20.9)	21.0 (20.1-22.0)
Lower estimated SES	130 (30.5)	181 (49.3)	172 (44.1)	160 (40.7)	152 (38.9)	154 (47.1)	146 (40.1)
Has a boyfriend	25 (5.7)	15 (4.0)	90 (23.0)	104 (26.4)	159 (40.4)	135 (41.3)	228 (62.5)
Sexually active	144 (33.4)	67 (17.7) ^d^	237 (60.5)	236 (59.9)	265 (67.8)	226 (69.1)	296 (81.1)
Coerced or tricked into sex ^e^	103 (23.9)	28 (7.4)	67 (17.4)	60 (15.2)	70 (17.8)	62 (19.0)	86 (24.0)
Engaged in transactional sex ^e^	51 (11.9)	18 (4.8)	46 (11.7)	36 (9.1)	52 (13.2)	71 (18.4)	63 (17.3)
Used a condom for sex	110 (85.6)	55 (91.7)	160 (74.4)	171 (77.0)	194 (84.7)	196 (85.6)	268 (82.7)
Currently pregnant: self-report	3 (2.3)	2 (0.5)	1 (0.3)	5 (1.3)	23 (9.4)	19 (8.4)	30 (8.2)
Ever been pregnant	15 (3.4)		16 (4.1)		55 (22.6)	60 (26.6)	114 (31.2)
Compared with pre–COVID-19, how has safety inside the home changed?							
Less safe					136 (34.5)	107 (27.9)	65 (17.8)
Same/no change					97 (24.6)	112 (29.2)	148 (40.6)
More safe					161 (40.9)	164 (42.8)	152 (41.6)
COVID-19 impact score							
Median (IQR)					10 (6-14)	10 (6-14)	10 (6-16)
Highest quartile					121 (30.7)	118 (30.7)	138 (37.8)
PHQ-9, mean (IQR)			2.87 (0-4)		2.39 (0-3)	2.10 (0-3)	
Elevated PHQ-9 score, ≥5			85 (21.7)		81 (20.6)	56 (17.1)	
BV: Nugent’s score, 7-10 ^f^	49 (11.2)	39 (9.2)	57 (14.4)	56 (14.1)	88 (22.2)	75 (22.8)	102 (28.0)
STI: composite of chlamydia, gonorrhea, and trichomoniasis	43 (9.9)		46 (11.6)		64 (16.2)		73 (20.1)
Proportion of STIs in which participant reports no sexual activity in past 6 mo, %	34.2		31.8		17.5		6.9

Abbreviations: BV, bacterial vaginosis; PHQ-9, 9-item Personal Health Questionnaire; SES, socioeconomic status; STI, sexually transmitted infection.

a Data are presented as No. (%) or median (IQR) unless noted otherwise. Blank cells indicate *not assessed.*

b Not all cells sum to the indicated sample size due to missing data. Missing data occurred in <1.6% of observations at any visit except at the 6-month visit, in which 47 surveys were lost due to a failed connection with the server.

c At the 36-month survey, 58 participants had relocated outside the study area and were followed only by telephone, thus without BV results.

d In abbreviating the 6-month survey from baseline, questions regarding sexual activity, transactional sex, and coerced sex were inadvertently removed, thus likely leading to a substantial underestimate. These questions were reintroduced from the 12-month survey onward.

e Coerced/tricked into having sex and transactional sex are among those who report being sexually active.

f Across all participants and visits, BV status was missing for 5 observations: 1 at 30 months, 2 at 37 months, and 2 at 48 months. All occurred in different individuals, all of whom had multiple observations of BV results in the pre– and post–COVID-19 periods and were therefore maintained in analyses.

Socioeconomic status (SES) at baseline was measured with questions related to household possessions, as reported by Zulaika et al.^24^ To reduce participant burden and for relevance to menstrual hygiene, only latrine type and water source used at home were asked at each subsequent 6-month visit and served as proxy for SES as a time-updated measure. Latrine type was scaled 1 to 4 points (bush/field, 1; traditional pit, 2; improved pit, 3; flush toilet, 4), and water source was scaled 1 to 4 points (surface water, 1; borehole, 2; rainwater, 3; piped water, 4) for a total of 8 points, with a higher score reflecting greater improvement. This proxy SES score was dichotomized at ≤3 (lower) vs 4 to 8 (higher). School-level WASH score (water, sanitation, and hygiene) at baseline was measured by previously described methods^23^; though schools were closed during the COVID-19 pandemic, this cluster-level variable was retained due to its relevance to area-level SES.

#### COVID-19–related stress

Stress related to the COVID-19 pandemic and mitigation measures was assessed at the 30-, 36-, and 48-month visits with 8 questions covering physical and emotional reaction domains related to COVID-19–specific psychological distress.^25^ These questions ask about changes in physical experiences (eg, sleeping, concentrating) and emotional ones (eg, feelings of anxiousness, worry). Response categories were simplified from 5 categories to *agree*, *don’t know/not sure*, and *disagree*. A COVID-19–related stress score was calculated by assigning 2 points per question if the participants agreed, 1 point if they responded *don’t know/not sure*, and 0 if they disagreed. The frequency distribution of responses to individual items over time is presented in Table S1. Cronbach’s alpha by visit was 0.87 (30 months), 0.86 (36 months), and 0.90 (48 months).

#### BV and STI testing and treatment

At baseline and each 6-month study visit, participants were asked to take self-collected vaginal swabs, a highly acceptable and validated approach for sample collection,^26–28^ to test for BV and STIs, as detailed previously.^20^ BV testing was done at each 6-month visit, and STI testing was conducted at baseline and 12-, 30-, and 48-month visits. Self-collected swabs for BV were immediately smeared on glass slides by the study staff and checked for sufficiency by a laboratory technician in the field. Slides were transported to the laboratory and gram stained, followed by Nugent’s scoring for detection of BV (score, 7-10).^29^ Infection with *Neisseria gonorrhoeae* and *Chlamydia trachomatis* were assessed via nucleic acid amplification test (GeneXpert; Cepheid) and *Trichomonas vaginalis* by rapid immunochromatographic assay (OSOM *T vaginalis* antigen detection assay; Sekisui). All participants were provided results, and those who tested positive for BV or STIs were offered antibiotic treatment, regardless of symptoms.

### Statistical analysis

This analysis had 2 components: (1) mixed effects modeling to quantify how BV and STI changed from the pre– to post–COVID-19 period and (2) longitudinal mediation analysis to explain how the relationship between COVID-19 stress and increased risk of BV was mediated. Due to minimal missing data, analyses were conducted as complete case.

#### Change in BV and STIs before vs during COVID-19

BV (Nugent’s score, 7-10 vs 0-6) is used as the primary outcome due to availability at all study visits (except the 24-month missed visit) and for high correlation with sexual activity.^21^ We examined the change in BV over time via generalized linear mixed effects models with Poisson distribution, with random effects for participant and cluster (school) and robust variance estimate. We examined the prevalence ratio of BV during the COVID-19 period (visits 30 through 48 months) as compared with pre–COVID-19 (baseline through 18 months), as well as multivariable models adjusted for intervention status and a priori confounders^20^: baseline STI status, SES, PHQ-9, and reports of having a boyfriend and engaging in transactional sex. As a secondary analysis, STIs were modeled by mixed effects models as just described. Mixed effects models were conducted in Stata/SE version 17 (StataCorp). In Kenya, as in many countries, sexual activity increases with age among adolescents and young adults,^30^ with the median age of sexual debut being 18.1 years nationally and 16.3 years for Siaya^31^; therefore, all models adjusted for age as a continuous variable, as an a priori confounder.^20^

#### Longitudinal mediation model

**Figure 1:**
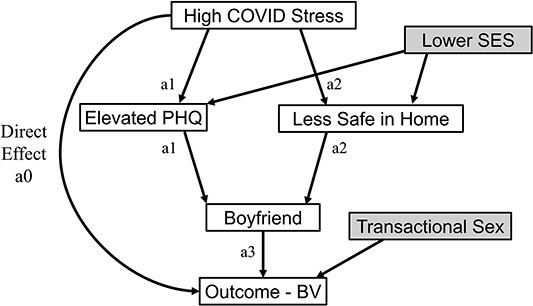
Conceptual model shows the hypothesized linkages between COVID-19–related stress and bacterial vaginosis (BV). The hypothesized relationship between elevated COVID-19–related stress and BV (direct effect, *a0*) is depicted with pathways being mediated through level 1 mediators (*a1*–elevated depressive symptoms [PHQ-9 ≥5] and *a2*–feeling less safe inside the home vs before the COVID-19 pandemic) and level 2 mediator *a3*–having a boyfriend. We hypothesized that socioeconomic status confounded the association between elevated COVID-19–related stress and increased depressive symptoms and feeling less safe inside the home. We also hypothesized that transactional sex confounded the association between elevated COVID-19–related stress and BV. Assigned intervention status (menstrual cup arm or control arm) and participant age were also controlled for and are not depicted in the figure. PHQ-9, 9-item Personal Health Questionnaire.

We conducted longitudinal mediation analysis to test our hypotheses that greater COVID-19–related stress mediated elevated depressive symptoms and feeling less safe inside the home, which in turn mediated the proximal exposure of having a boyfriend (Figure 1). The variable “sexually active” itself was not included in the model, since it is in the causal pathway for STIs and often for BV. The outcome for analysis was BV, as STI was measured only at annual study visits. We controlled for assigned intervention status as a known factor associated with BV,^20^ SES, and age as level 1 confounders and engaging in transactional sex as a level 2 confounder. The mediation approach differs from multivariable adjustment, as detailed in the previous section, in that the independent variable (COVID-19–related stress) is proposed to *influence* the mediator variables (depressive symptoms, domestic safety, having a boyfriend), which in turn influence the dependent variable (BV). In this way, the mediator variables seek to clarify *how* the independent and dependent variables are related.

The longitudinal mediation analysis decomposed the overall impact of higher COVID-19–related stress on the risk of getting BV into multiple distinct pathways via a counterfactual framework for mediation.^32^^,^^33^ We defined a generalized marginal structural model for nested counterfactuals where we directly parameterized each indirect effect pathway (Figure 1) through 4 distinct pathways: the direct effect and indirect pathways *a1*, *a2*, and *a3*. The *a1* pathway encompasses all potential pathways involving elevated depressive symptoms (PHQ-9 ≥5), while *a2* encompasses those associated with feeling less safe at home vs prior to the COVID-19 pandemic. Last, *a3* captures the influence of having a boyfriend on the outcome, BV. We derived parameter estimates of the direct and indirect effects by regressing BV on COVID-19–related stress (the exposure) as well as all the other possible pathways, using a weighted approach and incorporating random intercepts.^34^ Causal mediation analysis with a nonrare binary outcome often introduces some ambiguity due to the noncollapsibility of odds ratio, and we address this challenge using a log linear model with robust bootstrapping for SE estimation.^35^ We also report percentage of mediation effect. Longitudinal mediation was conducted in R (version 4.1.13).

## Results

Participants had a median age of 16.9 years at baseline and 21.0 years at the 48-month visit, in keeping with the 4-year span of time (Table 1). The proportion of participants reporting having a boyfriend and being sexually active increased over time, especially at the 12-, 30-, and 48-month visits. At the 30-month visit, when schools largely remained closed and curfews were still in place, over one-third (34.5%) of participants reported feeling less safe in their home vs before the COVID-19 pandemic and associated lockdowns; this decreased to 17.8% by the 48-month visit.

### Distribution of covariates by COVID-19–related stress

The median COVID-19 stress impact score was 10 at 30 months and remained similar across time points (Table S1). There were higher rates of agreement with statements related to worry about getting infected with COVID-19, friend and family getting infected with COVID-19, or transmitting COVID-19 to someone else, with the lowest rates of agreement with difficulty sleeping, concentrating, and feeling overwhelmed.

### Increase in BV and STIs in the COVID-19 period vs the pre–COVID-19 period

**Figure 2:**
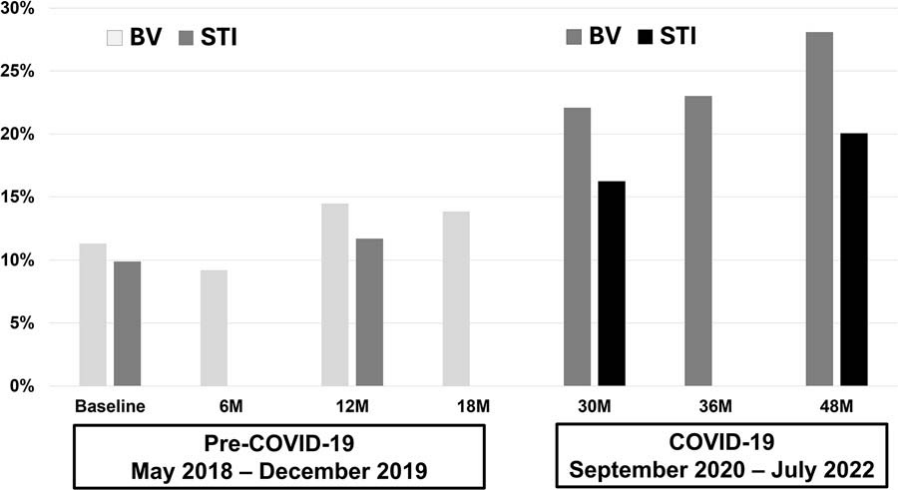
Prevalence of BV and STIs over time before and during COVID-19. The y-axis shows the prevalence of BV and STIs over study visits (x-axis). The pre–COVID-19 and COVID-19 periods are indicated with the dates underneath the x-axis with darker intensity of shading in the visits occurring during the COVID-19 period. BV, bacterial vaginosis; STI, sexually transmitted infection.

BV increased from 11.2% at baseline to 14.1% at 18 months and, in the COVID-19 period, to 22.2% at 30 months and 28.1% by 48 months (Figure 2). Similarly, STI prevalence rose from 9.9% at baseline to 11.6% at 12 months, rising to 16.2% at the 30-month visit and to 20.1% by the 48-month visit. At each time point during the COVID-19 period, the prevalence of BV was greater for participants with higher COVID-19–related stress, lower socioeconomic indicators, and elevated PHQ-9 scores, as well as reports of having a boyfriend, engaging in transactional sex, and feeling less safe inside the home (Table 2). In multivariable mixed effects modeling, there was a 26% higher relative prevalence of BV in the COVID-19 period vs the pre–COVID-19 period (adjusted prevalence ratio [aPR], 1.26; 95% CI, 1.00-1.59; Table 3), adjusted for assigned intervention status; baseline school-level WASH score, SES, sexual activity, and STI status; and time-varying age, SES, elevated PHQ-9 score, engaging in transactional sex, and having a boyfriend. Similarly, there was a 36% higher prevalence ratio of STI in the COVID-19 period as compared with the pre–COVID-19 period (aPR, 1.36; 95% CI, 0.98-1.88).

**Table 2:** Distribution of COVID-19–related stress and hypothesized mediating factors by time point and BV status.^a^

	**30 mo (n = 395)**		**36 mo (n = 327)**		**48 mo (n = 362)**	
**Variable**	**No BV (n = 307)**	**BV (n = 88)**	**No BV (n = 252)**	**BV (n = 75)**	**No BV (n = 260)**	**BV (n = 102)**
**Baseline covariates**						
Intervention arm						
Control	153 (75.7)	49 (24.3)	125 (73.5)	45 (26.5)	120 (65.9)	62 (34.1)
Cups	154 (79.9)	39 (20.2)	127 (80.9)	30 (19.1)	140 (77.8)	40 (22.2)
Socioeconomic status						
Higher	214 (77.3)	63 (22.7)	181 (79.4)	47 (20.6)	184 (72.7)	69 (27.3)
Lower	93 (78.8)	25 (21.2)	70 (71.4)	28 (28.6)	76 (69.7)	33 (30.3)
WASH score						
Lower (0-1)	129 (81.6)	29 (18.4)	95 (74.8)	32 (25.2)	103 (76.3)	32 (23.7)
Higher (2)	178 (75.1)	59 (24.9)	157 (78.5)	43 (21.5)	157 (69.2)	70 (30.8)
Sexually active						
No	208 (80.3)	51 (19.7)	171 (79.9)	43 (20.1)	182 (77.8)	52 (22.2)
Yes	96 (73.3)	35 (26.7)	78 (71.6)	31 (28.4)	75 (61.0)	48 (39.0)
Missing	3	2	3	1	3	2
STI status ^b^						
Negative	284 (79.4)	74 (20.7)	232 (78.6)	63 (21.4)	240 (73.6)	86 (26.4)
Positive	23 (62.2)	14 (37.8)	20 (62.5)	12 (37.5)	20 (55.6)	16 (44.4)
**Time-varying covariates**						
COVID-19–related stress						
Lower quartiles	216 (79.1)	57 (20.9)	171 (76.0)	54 (20.0)	168 (74.3)	58 (25.7)
Highest quartile	89 (74.2)	31 (25.8)	80 (79.2)	21 (20.8)	92 (67.6)	44 (32.4)
Missing	2		1			
Socioeconomic status						
Higher	193 (80.4)	47 (19.6)	140 (81.4)	32 (18.6)	158 (72.8)	59 (27.2)
Lower	112 (73.7)	40 (26.3)	111 (72.1)	43 (27.9)	102 (70.8)	42 (29.2)
Missing	2	1	1			
Age, y	19.5 (18.5-20.5)	19.3 (18.5-20.3)	19.8 (19.0-20.8)	20.2 (19.4-21.0)	20.9 (20.1-21.9)	21.2 (20.2-22.2)
Has a boyfriend						
No	195 (83.0)	40 (17.0)	167 (87.0)	25 (13.0)	112 (82.8)	25 (18.2)
Yes	110 (69.6)	48 (30.4)	84 (62.7)	50 (37.3)	148 (65.8)	77 (34.2)
Missing	2		1			
Engaged in transactional sex						
No	270 (79.2)	71 (20.8)	213 (79.2)	56 (20.8)	219 (73.2)	80 (26.7)
Yes	35 (67.3)	17 (32.7)	38 (66.7)	19 (33.3)	41 (65.1)	22 (34.9)
Missing	2		1			
Depressive symptoms						
PHQ-9 <5	248 (79.5)	60 (20.5)	209 (77.4)	61 (22.6)	211 (73.0)	78 (27.0)
PHQ-9 ≥5	57 (70.4)	24 (29.6)	42 (75.0)	14 (25.0)	43 (68.3)	20 (31.7)
Missing	2		1		6	4
Safety inside the home						
No change or more safe	207 (80.2)	51 (19.8)	186 (78.8)	50 (21.2)	217 (73.1)	80 (26.9)
Less safe	98 (72.6)	37 (27.4)	64 (71.9)	25 (28.1)	43 (66.1)	22 (38.9)
Missing	2		2			

Abbreviations: BV, bacterial vaginosis; PHQ-9, 9-item Personal Health Questionnaire; STI, sexually transmitted infection; WASH, water, sanitation, and hygiene.

a Data are presented as No. (%) or median (IQR).

b Composite of *Chlamydia trachomatis*, *Neisseria gonorrhoeae*, and *Trichomonas vaginalis*.

**Table 3:** Crude and multivariable adjusted mixed effects modeling: prevalence ratio of BV and STIs during COVID-19 vs pre–COVID-19.^a^

	**Prevalence ratio (95% CI)**	
	**Crude**	**Adjusted**
BV	N=2738	N=2544
COVID-19 vs pre–COVID-19	2.00 (1.62-2.48)	1.26 (1.00-1.59) ^b^
STIs, ^c^	N=1587	N=1511
COVID-19 vs pre–COVID-19	1.68 (1.40-2.02)	1.36 (0.98-1.88) ^d^

Abbreviations: BV, bacterial vaginosis; PHQ-9, 9-item Personal Health Questionnaire; SES, socioeconomic status; STI, sexually transmitted infection; WASH, water, sanitation, and hygiene.

a The pre–COVID-19 study visits of baseline through 18 months took place May 2018 through October 2019. Study visits at 30 through 48 months took place during the COVID-19 period, September 2020 through July 2022.

b Simultaneously adjusted for the following: assigned intervention status; baseline WASH score, SES, sexual activity, and STI status; and time-varying age, SES, PHQ-9 score, engaging in transactional sex, and having a boyfriend.

c Composite of testing positive for any of the following: *Chlamydia trachomatis*, *Neisseria gonorrhoeae*, and *Trichomonas vaginalis*.

d Simultaneously adjusted for the following: assigned intervention status; baseline WASH score, SES, and sexual activity; and time-varying age, SES, elevated PHQ-9 score, engaging in transactional sex, and having a boyfriend.

### Results of the mediation model

We hypothesized that stress related to COVID-19 could be mediating the increased prevalence of reproductive tract infection through depressive symptoms and domestic safety, both adversely affected by the pandemic and potentially related to higher likelihood of having a boyfriend and thus sexual exposure. To demonstrate the plausibility of the hypothesized relationships, the association between COVID-19–related stress and mediators was examined with mixed effects models. As shown in Table 4, feeling less safe inside the home vs before the COVID-19 pandemic (aPR, 2.16; 95% CI, 1.74-2.68) was elevated among participants with the highest-quartile COVID-19–related stress score, adjusted for numerous baseline and time-varying covariates, though elevated depressive symptoms were not. Adjusted for the same numerous baseline and time-varying confounders, having a boyfriend (level 2 mediator) was significantly higher with both level 1 mediators—elevated depressive symptoms (aPR, 1.20; 95% CI, 1.08-1.33) and feeling less safe inside the home as opposed to pre–COVID-19 (aPR, 1.21; 95% CI, 1.06-1.39)—but was not associated with COVID-19–related stress. In longitudinal mediation analysis, AGYW who reported higher COVID-19–related stress had a nonsignificant 11% increased prevalence ratio of BV (Table 5). Among the various pathways through which COVID-19–related stress could mediate the risk of BV, feeling less safe inside emerged as an important factor, contributing to 57.3% of the total effect. This was followed by the influence of having a boyfriend at 25.2% and elevated depressive symptoms at 15.3%.

**Table 4:** Crude and multivariable adjusted mixed effects modeling: prevalence ratios of level 1 and 2 mediators.^a^

	**Prevalence ratio (95% CI)**		
	**Crude**	**Adjusted for longitudinal mediation**	**Fully adjusted**
**Level 1 mediators**			
Elevated depressive symptoms: PHQ-9 ≥5	N=1143	N=1084	N=1069
Highest- vs lower-quartile COVID-19–related stress	1.24 (0.86-1.80)	1.25 (0.84-1.85) ^b^	1.11 (0.83-1.49) ^c^
Feels less safe inside the home vs before COVID-19	N=1142	N=1083	N=1069
Highest- vs lower-quartile COVID-19–related stress	2.28 (1.79-2.19)	2.27 (1.81-2.85) ^b^	2.16 (1.74-2.68) ^c^
**Level 2 mediator**			
Has a boyfriend	N=1143	N=1083	1069
Highest- vs lower-quartile COVID-19–related stress	1.16 (1.01-1.35)	1.02 (0.89-1.16) ^d^	0.98 (0.85-1.12) ^e^
Elevated depressive symptoms: PHQ-9 ≥5		1.20 (1.01-1.41) ^d^	1.20 (1.08-1.33) ^e^
Feels less safe inside the home during COVID-19 vs before		1.22 (1.03-1.45) ^d^	1.21 (1.06-1.39) ^e^

Abbreviations: PHQ-9, 9-item Personal Health Questionnaire; SES, socioeconomic status; STI, sexually transmitted infection; WASH, water, sanitation, and hygiene.

a All models, including crude models, are time adjusted. Level 1 mediators are association with elevated COVID-19–related stress. Level 2 mediator is evaluated in association with COVID-19–related stress and level 1 mediators.

b Simultaneously adjusted for assigned intervention status and time, as well as level 1 confounders (time-varying SES and age).

c Simultaneously adjusted for the following: assigned intervention status; baseline WASH score, SES, sexual activity, and STI status; time and time-varying age, SES, elevated PHQ-9 score (for safety inside the home model), feeling less safe inside the home (for depressive symptoms model), engaging in transactional sex, and having a boyfriend.

d Simultaneously adjusted for the following: assigned intervention status and time, level 1 confounders (time-varying SES and age), level 1 mediators (depressive symptoms, feeling safe inside the home), and level 2 confounder (transactional sex).

e Simultaneously adjusted for the following: assigned intervention status; baseline WASH score, SES, sexual activity, and STI status; time and time-varying age, SES, elevated PHQ-9 score, feeling safe inside home, and engaging in transactional sex.

**Table 5:** Crude and variable adjusted longitudinal pathway model: prevalence ratio of BV for adolescent girls and young women with higher COVID-19–related stress.^a^

	**PR estimate (95% CI)** ^**b**^		
	**Crude model**	**Longitudinal pathway model**	**Mediation, %**
**Direct effect**			
Elevated COVID-19–related stress and BV	1.11	1.00 (0.91-1.09)	2.2
**Indirect effect**			
*a1:* association with BV mediated through elevated depressive symptoms (PHQ-9 ≥5)		1.02 (0.94-1.10)	15.3
*a2:* association with BV mediated through feeling less safe inside the home vs before COVID-19		1.06 (0.98-1.14)	57.3
*a3:* association with BV mediated through having a boyfriend		1.03 (0.95-1.11)	25.2
**Total effect**	1.11	1.11	100

Abbreviations: BV, bacterial vaginosis; PHQ-9, 9-item Personal Health Questionnaire.

^a^ The longitudinal mediation analysis decomposes the overall impact of higher COVID-19–related stress vs less stress on the risk of having BV, following relationships as hypothesized in Figure 1 (*a1*–*a3*).

^b^ 95% CI based on bootstrapped SE.

## Discussion

In this cohort of AGYW in western Kenya, the prevalence of BV and STIs rose over time, with greater increases observed during the period of COVID-19, immediately following school closures. The data captured in our cohort with time accruing before and during the COVID-19 pandemic enabled us to quantify this increase and evaluate reasons behind it. AGYW who were more greatly affected by the pandemic, as measured by COVID-19–related stress, were also more likely to report feeling less safe inside the home and in turn were more likely to have a boyfriend, which was proximal to having BV. Despite the association of these factors with greater COVID-19–related stress and with BV and counter to our hypothesis, the direct effect of COVID-19–related stress on BV was small and nonsignificant, indicating that the greater likelihood of BV was due to the constellation of factors that were affected during the COVID-19 pandemic.

Having a boyfriend, the most proximal factor to BV and having greater likelihood of sexual exposure, may represent a coping mechanism in response to financial stress, feeling less safe in the home, or feelings of depression and anxiety. As reviewed in a study of sexual coping mechanisms during the COVID-19 pandemic, sexual connections and the intimacy entailed “can help cope with stressors and traumatic events.”^36^ While not disaggregated by gender, the most frequently reported sexual strategies were sex as a source of pleasure and intimacy, to bond with and please a partner, to express care and strengthen the relationship, or to relieve stress and relax. A cross-sectional survey of Kenyan schoolgoing adolescents aged 13 to 19 years living in Nairobi or the coast region found high prevalences of anxiety (19.1%) and elevated depressive symptoms (19.1% with PHQ-9 *>*10), as well as high mean scores for emotional and behavioral problems.^37^

Mbithi et al also observed that living in unsafe neighborhoods, being physically forced to have sex, and drinking alcohol were associated with higher odds of depressive symptoms and anxiety. In a mobile phone–based survey of 2224 pairs of adults and adolescents (age, 10-19 years) from Kisumu, Nairobi, and the coastal region, 36% of adolescents reported depressive symptoms, and this was associated with adult loss of income, which in turn was associated with household tensions and violence.^38^ We observed marginal significance of elevated depressive symptoms among those with higher COVID-19–related stress, and there are other dimensions of mental health that we did not measure. As we continue follow-up of these AGYW, we have incorporated expanded measures of stress and anxiety. Our qualitative work will incorporate questions related to what extent and how sexual relationships may be serving as a coping response to stress and how these AGYW respond to and cope with stress more broadly. This may provide insight for future interventions to provide AGYW with healthy coping strategies.

Alternatively, having a boyfriend may represent increased sexual exposure through financial need or coercion. Our qualitative study involving focus groups with study participants and men in their communities provides further insight.^39^ AGYW expressed that there were elevated drivers for sex during the COVID-19 lockdowns, school closures, and restrictions: increased poverty with concomitant pressures for transactional sex, from men, peers, and parents; boredom and freedom to visit boyfriends; COVID-19 restrictions and curfews that left them having to spend nights with boyfriends; and greater competition for boyfriends, leading to more risk taking (eg, having sex). Men expressed that providing material and financial support was helping AGYW during the pandemic, and at the same time they had awareness that this enabled them a power differential in engaging girls to have sex or to have sex without condoms. In semistructured in-depth interviews with 34 adolescent-caregiver dyads who had participated in the Shamba Maisha agricultural microfinance intervention in Kenya, AGYW receiving the intervention reported “no longer needing to engage in transactional sex or have multiple concurrent sexual partners as a way to meet their basic needs.”^40^ Interviews revealed that girls felt less pressure for reciprocating for assistance with sex and that power differentials were reduced by not having to rely on men for food and other needs. Although the Shamba Maisha intervention took place prior to the pandemic, structural interventions addressing underlying drivers of sexual risk taking may sustainably reduce AGYW vulnerability to economic disruptions, such as those brought by the COVID-19 pandemic.

Feeling less safe in the home vs before the COVID-19 pandemic was elevated among AGYW reporting higher levels of COVID-19–related stress and was associated with a higher prevalence of BV. As noted, there were worldwide increases in all forms of violence against women and girls (including domestic violence, gender-based violence, and sexual assault), and this has been named the “shadow pandemic” by UN Women.^41^ The conditions of the pandemic, especially during initial lockdowns and longer-lasting restrictions on movement, led to financial stress, isolation with abusers, and tighter living quarters, as well as reduced access to health care, police and justice, and social services. UN Women called for national responses that provide psychosocial support to survivors, expanded services (eg, shelters, hotlines, counseling), and “strong messages” that violence against women and girls will be met with legal response.^42^ The COVID-19 pandemic demonstrated the need and opportunity for virtual applications to provide services,^43^ though the applicability of virtual services remains challenging for rural and other areas with preexisting limited infrastructural resources for technology and confidentiality, as well as limited or nonexistent access to in-person services. While we did not measure whether feeling less safe was physical, emotional, or sexual, results such as ours reflecting the downstream impacts of domestic safety on the reproductive health of AGYW highlight the need to invest in and ensure that these services are functionally in place prior to or shortly following the next population-level crisis. Interventions that are more likely to have a significant and sustained impact on preventing gender-based violence are not quick or easy solutions, such as community- and school-based promotion of gender equality, transforming gender stereotypes and discriminatory norms, reforming discriminatory laws, ensuring women’s access to formal wage employment and education, and increasing women’s access to financial security.^44^ As Mehta and Seeley have written,^45^ the progress in sexual and reproductive health of the past 30 years is at critical juncture due to ongoing population-level crises of pandemics, climate change, and conflict.

### Limitations

The longitudinal multilevel mediation analysis with multiple mediators at each level is quite complex, particularly when dealing with binary as opposed to continuous mediators and outcomes. With limited work in this area, defining an appropriate model with assumptions that satisfies all the data constraints is challenging. Marginal structural modeling is an effective method when it comes to modeling sequential mediators, but there is limited work that implements time-varying multilevel mediation. For this reason, we were selective in our choice of mediators. Subsequent analyses will examine whether BV and STI risks changed in the post–COVID-19 period. We believe that our findings may generalize to AGYW who attended secondary school and live in similar setting, but the impacts of the COVID-19 pandemic on reproductive tract infections and drivers of this among those who did not attend school or live in different settings may be different.

## Conclusions

In this cohort of AGYW, the prevalence of BV and STIs increased following school closures during the COVID-19 pandemic. Longitudinal analysis demonstrated that this higher risk was mediated by depressive symptoms and feeling less safe in the home, which led to a greater likelihood of having boyfriends and thus sexual exposures. These results highlight specific modifiable factors that can be targeted by interventions during crises to help maintain sexual and reproductive health resiliency in AGYW: mitigating mental health impacts and domestic safety concerns. Our results also indicate that further research is needed to elucidate the benefits and risks of relying on boyfriends and sex partners to cope with these stressors and how these relationships may be leveraged as a resource.

## Supplementary Material

Cache2_Supplementary_Material_20Mar24_qfae045
