# Supplementary material for: Increased reproductive tract infections among secondary school girls during the COVID-19 pandemic: associations with pandemic-related stress, mental health, and domestic safety
Source: Sex Med. 2024 Jul 23;12(3):qfae045. doi: 10.1093/sexmed/qfae045 (PMC11264406; doi:10.1093/sexmed/qfae045)
Supplement: Cache2_Supplementary_Material_20Mar24_qfae045 [file cache2_supplementary_material_20mar24_qfae045.docx]

Supplementary Material

Increased reproductive tract infections among secondary school girls during the COVID-19 pandemic: associations with pandemic related stress, mental health, and domestic safety

**Supplemental Table 1. Distribution of responses to COVID-19 related stress questions^1^ by study time point.**

| **Please tell us whether you agree, disagree, or don’t know for the following statements.** | **30 Month visit, N=394**  **n (%)** | **36 Month visit, N=327**  **n (%)** | **48 Month visit, N=365**  **n (%)** |
| --- | --- | --- | --- |
| **I am very worried about getting the coronavirus/COVID**  Agree  Disagree  Don’t know | 306 (77.7)  57 (14.5)  31 (7.9) | 259 (79.2)  55 (16.8)  13 (4.0) | 272 (74.5)  75 (20.6)  18 (4.9) |
| **I am very worried about my family or friends getting the coronavirus/COVID**  Agree  Disagree  Don’t know | 312 (79.2)  54 (13.7)  28 (7.1) | 267 (81.6)  45 (13.8)  15 (4.6) | 279 (76.4)  68 (18.3)  18 (4.9) |
| **I am very worried about giving someone else the coronavirus/COVID**  Agree  Disagree  Don’t know | 286 (72.6)  78 (19.8)  30 (7.6) | 234 (71.6)  74 (22.6)  19 (5.8) | 256 (70.1)  88 (24.1)  21 (5.8) |
| **I have had a hard time sleeping because of the coronavirus/COVID**  Agree  Disagree  Don’t know | 131 (33.2)  239 (60.7)  24 (6.1) | 107 (32.7)  200 (61.2)  20 (6.1) | 141 (38.6)  195 (53.4)  29 (8.0) |
| **I have had difficulties concentrating because of the coronavirus/COVID**  Agree  Disagree  Don’t know | 150 (38.1)  215 (54.6)  29 (7.4) | 132 (40.4)  179 (54.7)  16 (4.9) | 148 (40.5)  190 (52.1)  27 (7.4) |
| **Thinking about the coronavirus/COVID makes me anxious**  Agree  Disagree  Don’t know | 224 (56.8)  146 (37.1)  24 (6.1) | 191 (58.4)  120 (36.7)  16 (4.9) | 205 (56.2)  134 (36.7)  26 (7.1) |
| **I am feeling overwhelmed by the coronavirus/COVID**  Agree  Disagree  Don’t know | 174 (44.2)  177 (44.9)  43 (10.9) | 126 (38.5)  169 (51.7)  32 (9.8) | 159 (43.6)  170 (46.6)  36 (9.9) |
| **I am worried about money because of the coronavirus/COVID**  Agree  Disagree  Don’t know | 246 (62.4)  126 (32.0)  22 (5.6) | 224 (68.5)  93 (28.4)  10 (3.1) | 256 (70.1)  94 (25.7)  15 (4.1) |

^1^ COVID-19 related distress adapted from [25].

**Figure 1. Timeline of CaCHe Study Visits.**


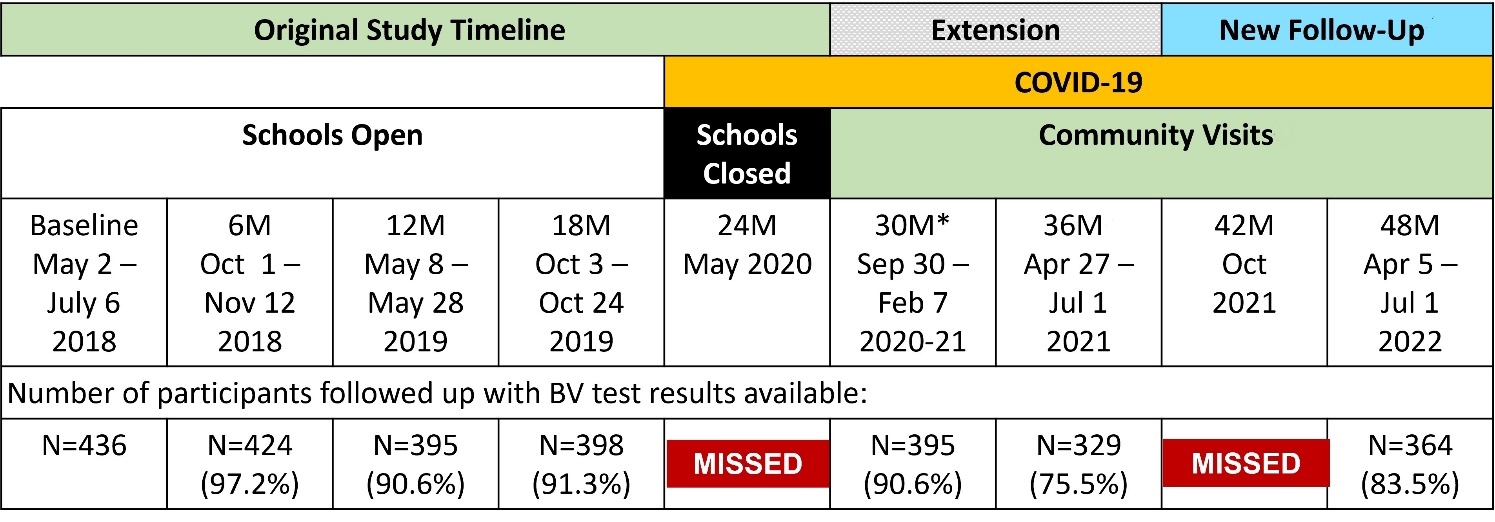


**Legend:** Baseline began May 2018, and study visits took place at schools every 6 months through October 2019 (18-month visit). The 24-month planned endline visit was cancelled due to COVID-19 restrictions and school closures. As schools were closed, we conducted the 30-month visit in the community, as were subsequent study visits due to participants completing school. With careful rebudgeting, we extended follow-up to 36-months, to continue capturing COVID-19 related impacts. At the 36-month visit, specimens were obtained from 329 participants; an additional 58 participants who had relocated were followed-up by telephone as the funding available did not support travel outside the area. Subsequent funding allowed for follow-up for all participants, including those who left the study area. *Due to COVID-19 precautions which limited the number of persons able to gather, the study period for the 30-month visit was extended, with 372 participants completed between September 30 – December 18, 2020, and an additional 25 participants between January 15 – February 7, 2021.

**STROBE Statement—checklist of items that should be included in reports of observational studies**

|  | Item No | Recommendation | Page number |
| --- | --- | --- | --- |
| **Title and abstract** | 1 | (*a*) Indicate the study’s design with a commonly used term in the title or the abstract | Abstract |
|  |  | (*b*) Provide in the abstract an informative and balanced summary of what was done and what was found | Abstract |
| Introduction | | |  |
| Background/rationale | 2 | Explain the scientific background and rationale for the investigation being reported | Background  Pages 4-5 |
| Objectives | 3 | State specific objectives, including any prespecified hypotheses | Background  Page 5  paragraph 4 |
| Methods | | |  |
| Study design | 4 | Present key elements of study design early in the paper | Methods  Page 6  “Study Design and Participants” |
| Setting | 5 | Describe the setting, locations, and relevant dates, including periods of recruitment, exposure, follow-up, and data collection | Methods  Pages 6-7 |
| Participants | 6 | (*a*) *Cohort study*—Give the eligibility criteria, and the sources and methods of selection of participants. Describe methods of follow-up  *Case-control study*—Give the eligibility criteria, and the sources and methods of case ascertainment and control selection. Give the rationale for the choice of cases and controls  *Cross-sectional study*—Give the eligibility criteria, and the sources and methods of selection of participants | Methods  Pages 6-7 |
|  |  | (*b*) *Cohort study*—For matched studies, give matching criteria and number of exposed and unexposed  *Case-control study*—For matched studies, give matching criteria and the number of controls per case | NA |
| Variables | 7 | Clearly define all outcomes, exposures, predictors, potential confounders, and effect modifiers. Give diagnostic criteria, if applicable | Methods  Pages 8-12 |
| Data sources/ measurement | 8 | For each variable of interest, give sources of data and details of methods of assessment (measurement). Describe comparability of assessment methods if there is more than one group | Methods  Pages 7-10 |
| Bias | 9 | Describe any efforts to address potential sources of bias | Methods  Pages 10-12 |
| Study size | 10 | Explain how the study size was arrived at | Methods  Page 6 |
| Quantitative variables | 11 | Explain how quantitative variables were handled in the analyses. If applicable, describe which groupings were chosen and why | Methods  Pages 7-12 |
| Statistical methods | 12 | (*a*) Describe all statistical methods, including those used to control for confounding | Methods  Pages 10-12 |
|  |  | (*b*) Describe any methods used to examine subgroups and interactions | NA |
|  |  | (*c*) Explain how missing data were addressed | Methods  Page 8, 10 |
|  |  | (*d*) *Cohort study*—If applicable, explain how loss to follow-up was addressed | Methods  Page 7-8 |
|  |  | (*e*) Describe any sensitivity analyses | NA |
| Results | | | |
| Participants | 13 | (a) Report numbers of individuals at each stage of study—eg numbers potentially eligible, examined for eligibility, confirmed eligible, included in the study, completing follow-up, and analysed | Figure 1, and throughout tables |
|  |  | (b) Give reasons for non-participation at each stage | NA |
|  |  | (c) Consider use of a flow diagram | Figure 1 |
| Descriptive data | 14 | (a) Give characteristics of study participants (eg demographic, clinical, social) and information on exposures and potential confounders | p. 13 and Table 1 |
|  |  | (b) Indicate number of participants with missing data for each variable of interest | Tables 1 and 2 |
|  |  | (c) *Cohort study*—Summarise follow-up time (e.g., average and total amount) | Figure 1 and Table 1 |
| Outcome data | 15 | *Cohort study*—Report numbers of outcome events or summary measures over time | Table 1 |
|  |  | *Case-control study—*Report numbers in each exposure category, or summary measures of exposure | NA |
|  |  | *Cross-sectional study—*Report numbers of outcome events or summary measures | NA |
| Main results | 16 | (*a*) Give unadjusted estimates and, if applicable, confounder-adjusted estimates and their precision (eg, 95% confidence interval). Make clear which confounders were adjusted for and why they were included | Tables 3, 4, 5 summarized in pp. 13-14 |
|  |  | (*b*) Report category boundaries when continuous variables were categorized | NA |
|  |  | (*c*) If relevant, consider translating estimates of relative risk into absolute risk for a meaningful time period | NA |
| Other analyses | 17 | Report other analyses done—eg analyses of subgroups and interactions, and sensitivity analyses | NA |
| Discussion | | | |
| Key results | 18 | Summarise key results with reference to study objectives | pp. 15-16 |
| Limitations | 19 | Discuss limitations of the study, taking into account sources of potential bias or imprecision. Discuss both direction and magnitude of any potential bias | pp.19 |
| Interpretation | 20 | Give a cautious overall interpretation of results considering objectives, limitations, multiplicity of analyses, results from similar studies, and other relevant evidence | pp. 15-19 |
| Generalisability | 21 | Discuss the generalisability (external validity) of the study results | pp.19 |
| Other information | | | |
| Funding | 22 | Give the source of funding and the role of the funders for the present study and, if applicable, for the original study on which the present article is based | Title page |
